# Cardiac telocytes exist in the adult *Xenopus tropicalis* heart

**DOI:** 10.1111/jcmm.14947

**Published:** 2020-01-12

**Authors:** Luocheng Lv, Zhaofu Liao, Jiali Luo, Hongyi Chen, Hongyan Guo, Jifeng Yang, Ruijin Huang, Qin Pu, Hui Zhao, Ziqiang Yuan, Shanshan Feng, Xufeng Qi, Dongqing Cai

**Affiliations:** ^1^ Key Laboratory of Regenerative Medicine Ministry of Education Jinan University Guangzhou China; ^2^ Joint Laboratory for Regenerative Medicine Chinese University of Hong Kong‐Jinan University Guangzhou China; ^3^ International Base of Collaboration for Science and Technology (JNU) Ministry of Science and Technology, Guangdong Province Guangzhou China; ^4^ Department of Developmental and Regenerative Biology Jinan University Guangzhou China; ^5^ Department of Neuroanatomy Institute of Anatomy University of Bonn Bonn Germany; ^6^ Department of Anatomy and Molecular Embryology Institute of Anatomy and Cell Biology University of Freiburg Freiburg Germany; ^7^ Stem Cell and Regeneration TRP School of Biomedical Sciences Chinese University of Hong Kong Hong Kong China; ^8^ Department of Medical Oncology Cancer Institute of New Jersey Robert Wood Johnson of Medical School New Brunswick NJ USA

**Keywords:** cardiac telocytes, regeneration of cardiomyocytes, *Xenopus tropicalis*

## Abstract

Recent research has revealed that cardiac telocytes (CTs) play an important role in cardiac physiopathology and the regeneration of injured myocardium. Recently, we reported that the adult *Xenopus tropicalis* heart can regenerate perfectly in a nearly scar‐free manner after injury via apical resection. However, whether telocytes exist in the *X tropicalis* heart and are affected in the regeneration of injured *X tropicalis* myocardium is still unknown. The present ultrastructural and immunofluorescent double staining results clearly showed that CTs exist in the *X tropicalis* myocardium. CTs in the *X tropicalis* myocardium were mainly twined around the surface of cardiomyocyte trabeculae and linked via nanocontacts between the ends of the telopodes, forming a three‐dimensional network. CTs might play a role in the regeneration of injured myocardium.

## 1 BACKGROUND

Regeneration of the damaged mammalian myocardium is a major challenge in clinical settings. After cardiac injury, such as myocardial infarction (MI), adult humans and non‐human mammals show very limited regenerative ability to replace the lost cardiomyocytes, as adult mammalian cardiomyocytes have very low capacity for cell proliferation and division. Necrotic cardiomyocytes are replaced with scar tissue, impairing the contractility of the remaining myocardium and even resulting in heart failure and death if the damage is severe.^1^ Thus, regeneration of the damaged myocardium is pursued as a therapeutic goal.

Recent studies have revealed that stromal cells communicate responsiveness to physiopathological stimuli through continuous bidirectional crosstalk between cardiomyocytes and noncardiomyocytes, such as cardiac fibroblasts, endothelial cells and cardiac telocytes (CTs), which act as ‘cardiovascular units’ (CVUs) and functional and structural building blocks of the heart to maintain the integrity of myocardial function.^2^, ^3^, ^4^, ^5^, ^6^, ^7^ During development and under physiopathological conditions, cardiac stromal cells and endothelial cells control the proliferation, growth and differentiation of cardiomyocytes in the myocardium.^8^, ^9^, ^10^ One important discovery is the identification of a novel type of stromal cell named telocytes, which are found in humans and rodents in the interstitium of the heart,^7^, ^11^, ^12^, ^13^, ^14^, ^15^, ^16^, ^17^ skeletal muscle,^18^ trachea and lung,^19^, ^20^ intestine,^21^ uterus and fallopian tubes,^22^ placenta^23^ and mammary glands^24^ and in the interstitium of the leech *Hirudo medicinalis*.^25^ CTs were found to be niche supporting cells that nurse cardiac stem cells and other cardiac cells in the myocardium and play an important role in regeneration following MI.^26^ Recently, we reported that the death of CTs is an important mechanism that contributes to the structural damage and poor healing and regeneration observed in MI.^27^, ^28^, ^29^ This evidence reveals that CTs provide a unique structural and functional microenvironment for maintaining the integrity of the myocardium and the regeneration of damaged myocardium.

Lower vertebrates, such as newts and zebrafish, display an extraordinary ability of cardiac tissue regeneration.^30^, ^31^, ^32^ Among anurans (frogs and toads), it is known that frog tadpoles can regenerate their tails,^33^ and adult *Xenopus* has a high capacity for retinal regeneration.^34^, ^35^ Recently, we reported for the first time that the adult *Xenopus tropicalis* heart can regenerate perfectly in a nearly scar‐free manner after injury via apical resection.^36^ However, whether telocytes exist in the *X tropicalis* heart and are affected in the regeneration of injured *X tropicalis* myocardium is still unknown. This study is designed to investigate this intriguing issue.

## 2 MATERIALS AND METHODS

### 2.1 Experimental animals

*Xenopus tropicalis* frogs (Nigerian strain) were purchased from NASCO (USA) and maintained in a freshwater tank at 26°C under a 12‐hour/12‐hour light/dark cycle. All the experimental protocols related to *X tropicalis* were approved by the Jinan University Animal Care Committee.

### 2.2 Collection of *Xenopus tropicalis* heart

**Figure 1:**
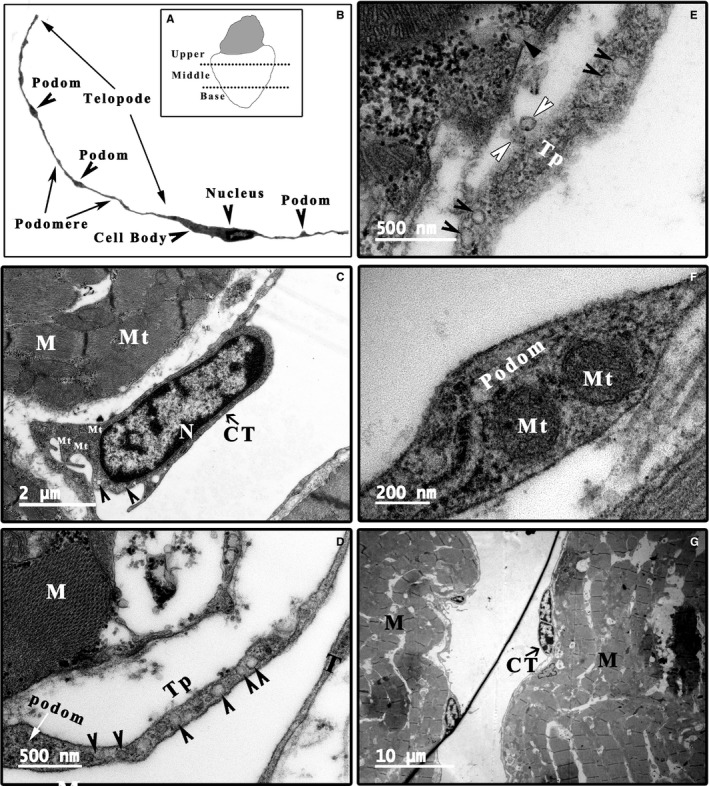
TEM analysis of the morphology of CTs in the *Xenopus tropicalis* heart. A, Schematic of the upper region, middle region and base of the *X tropicalis* heart for TEM analysis. B, Representative CTs with a hallmark ultrastructural morphology: a thin perinuclear rim of cytoplasm with few organelles and thin cytoplasmic veils containing mitochondria. Long telopodes (up to 100 μm), which represent cellular prolongations of the telocytes with moniliform (segments approximately 100 nm thick, named podoms) processes. C, A representative CT cell body (arrowhead: microvesicle). D, A representative telopode with podom (white arrow: podom; arrowhead: microvesicle). E, A representative telopode with many microvesicles (arrowhead) and secreted microvesicle (white arrowhead). F, A representative podom containing mitochondria. G, CTs in mitosis. CT: Cardiac telocyte; M: Cardiomyocyte; Mt: Mitochondria; N: Nucleus; Scale bar: Size as shown in the figures; Tp: Telopode

*Xenopus tropicalis* frogs (4 females; 12 months old) were used in the present study. Representative sections of the upper region, middle region and base of the individual ventricles (Figure 1A) were collected for transmission electron microscopy (TEM) analysis.

### 2.3 Apical resection of the *Xenopus tropicalis* heart

Apical resection of the *X tropicalis* heart was performed based on our recently established protocol.^36^ Briefly, *X tropicalis* frogs were placed in a tricaine methanesulfonate (MS‐222; 1 mg/mL; TCI) bath that was prepared with sterile double‐distilled water at room temperature for 4 minutes, incubated on ice for 60 seconds and then positioned ventral side up on an ice pad. The skin of the chest and upper abdomen was sterilized with iodine and 75% alcohol. A small incision was made near the heart using ophthalmic scissors. The pericardial sac was then opened, and the ventricle was exposed. Approximately 10% (approximately 1 mm in length) of the ventricle tissue from the cardiac apex was resected with Vannas scissors (Figure 6A). The opened cavity was sutured with 4‐0 suture after amputation. The animals were subsequently transferred to and maintained in freshwater at 26°C. The injured hearts were collected at 2 or 8 days after apical resection (daar). A cross‐section (approximately 1.5 mm) that included the wound zone was collected at 2 or 8 daar for TEM (Figure 6A).

### 2.4 Transmission electron microscopy

The samples from cross‐sections of *X tropicalis* heart were fixed in a solution of 1% osmium tetroxide and 1.25% potassium ferrocyanide for 30 minutes at room temperature. After washing in PBS (pH 7.2) for 5 minutes at room temperature, specimens were immersed overnight in 0.1% osmium tetroxide in PBS at room temperature and then processed for TEM observation.

### 2.5 Semiquantitative analysis of CTs

ImageJ version 1.48 was used to measure and analyse the CT cell bodies, telopodes, podoms, contacts, vesicles and caveolae. The longest and shortest diameters of vesicles, caveolae, CT cell bodies, CT nuclei and CT podoms; gaps between a CT and a cardiomyocyte; and gaps between a telopode and a cardiomyocyte were measured. The values are presented as the means ± standard deviation (SD). The counting numbers for all above‐observed parameters are listed in Tables S1 and S2.

### 2.6 Immunohistochemistry for CTs

The cryo‐section of the collected *X tropicalis* hearts (8 μm) was kept at room temperature for 30 minutes, washed for three times with PBS (pH = 7.4; each for 3 minutes) and then post‐fixed with 4% Paraformaldehyde for 30 minutes. After three wash with PBS, the sections were permeabilized and blocked with PBS containing 0.5% Triton X‐100 and 1% bovine serum albumin (BSA) at room temperature for 60 minutes. The sections were then successively incubated overnight at 4°C with a combination of the following antibodies: goat anti–c‐Kit (1:100; Cat No. sc‐168, Santa Cruz Biotechnology)/rabbit anti‐CD31 (1:100; Cat No. NB100‐2284, NOVUS), or goat anti–c‐Kit (1:100; Cat No. sc‐168, Santa Cruz Biotechnology)/rabbit anti‐vWF (1:100; Cat No. F3520, Sigma‐Aldrich), diluted in PBS containing 1% BSA and 0.05% Triton X‐100. After three wash with PBS, the sections were incubated for 1 hour at room temperature with a combination of secondary antibodies: donkey anti‐goat Alexa 555 (1:100; Cat No. A‐21432, Invitrogen) and donkey anti‐rabbit Alexa 488 (1:100; Cat No. A‐21206, Invitrogen). The sections were then subsequently counterstained with DAPI and mounted with mounting medium. The images were captured using confocal microscope (Carl Zeiss: LSM 880, Microimaging Inc).

## 3 RESULTS

### 3.1 Identification and characterization of CTs in the *Xenopus tropicalis* heart

**Figure 2:**
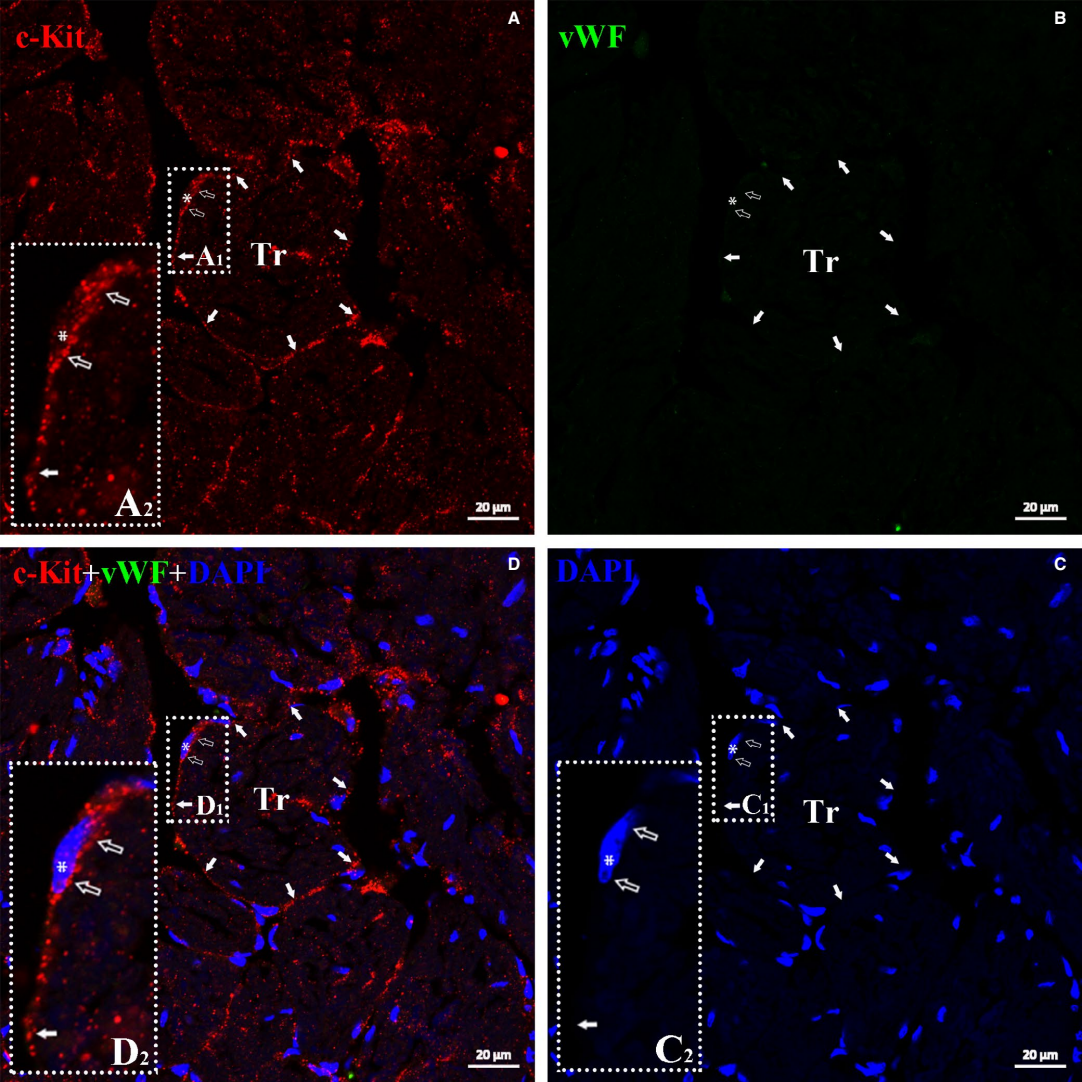
TEM identified CTs in myocardium is c‐Kit–positive but vWF‐negative. Double immuofluorescent staining for anti–c‐Kit (red) and vWF (green) demonstrated c‐Kit^+^ and vWF^‐^ cells with very small cell bodies (open arrow), a nucleus (approximately 1:1 ratio of the cytoplasm to the nucleus; white asterisk) and extremely thin prolongation (telopode) around trabeculae in the *Xenopus tropicalis* myocardium (white arrow). Showing that TEM identified CTs express c‐Kit, a generally accepted marker of CTs, but not vWF, a unique marker of endothelial cell. A, Anti–c‐Kit (red); B, Anti‐vWF (green); C, DAPI; D, merged of A, B and C. A_2_, C_2_ and D_2_ are higher magnification of A_1_, C_1_ and D_1_, respectively, which are showing the cell body and part of telopode of CTs. Scale bar: 20 μm. Tr: Trabecula of the myocardium

TEM examination is fundamental for identifying telocytes according to their hallmark ultrastructural cellular compartments: (a) a cell body, (b) cellular prolongations (telopodes) and (c) the labyrinthine network composed of telopodes. A typical telocyte has a thin perinuclear rim of cytoplasm with few organelles such as mitochondria. Telopodes, unique morphological structures of telocytes, are the longest structures in the body (up to 100 μm), with a moniliform appearance (approximately 100 nm thick, named podoms) displaying a dichotomous branching pattern. Caveolae, coated vesicles, mitochondria, well‐developed smooth and rough endoplasmic reticula, thin filaments and microtubules are present in the podoms of telopodes. Another distinctive ultrastructural feature of telopodes is the formation of labyrinthine networks by three‐dimensional convolutions and cytoplasmic overlapping. All these unique characteristics make these cells completely different from other types of myocardial stromal cells.^5^, ^6^, ^7^, ^37^ Accordingly, TEM analysis was performed to identify CTs in the *X tropicalis* heart. The representative dimensions of the upper region, middle region and base of the *X tropicalis* heart were investigated. Many cells that had a small and long ovoid cell body with the scarce cytoplasm occupied by a large nucleus and elements of the cytoskeleton and thin and very long telopodes with moniliform podomeres were visualized in the myocardium of the upper region, middle region and base region of ventricle (Figure 1B‐G). In addition, many microvesicles and mitochondria were found in the telopodes and podoms (Figure 1D‐F). Some CTs were found undergoing mitosis (Figure 1G). To date, a unique marker for CTs has not yet been identified, while c‐Kit, CD34 and PDGF‐Rα are generally applied as markers to identify CTs in histological scenarios.^27^, ^28^, ^29^, ^38^, ^39^, ^40^ Therefore, c‐Kit was applied as a marker to conduct immunofluorescent staining to identify the CTs in the present study. To double confirm that the identified cell was a CT and not another cell type, such as endothelial cells, whose morphology is similar to that of the identified CTs in *X tropicalis* myocardium, double immunofluorescent staining for anti–c‐Kit combined with anti‐CD31 or anti‐vWF, unique markers for endothelial cells, was conducted in the representative *X tropicalis* myocardium. Both anti–c‐Kit and anti‐CD31, as well as anti–c‐Kit and anti‐vWF double immunofluorescent staining, demonstrated that the CT identified by TEM not only possessed the unique morphology of CTs but also was c‐Kit–positive and CD31‐ and vWF‐negative (Figure 2; Figures [Link], [Link], [Link]). This confirms that the identified CTs by TEM were not endothelial cells.

Semiquantitative analysis of CTs in the *X tropicalis* myocardium was performed. The longest diameter of the CT cell bodies was 7.68 ± 1.82 μm, while the shortest diameter was 2.00 ± 0.64 μm. The longest diameter of the CT nuclei was 5.84 ± 1.73 μm, while the shortest diameter was 1.64 ± 0.60 μm. The ratio of the area of the nucleus to the area of the cell body was 0.64 ± 0.14. The length of the telopodes was 19.65 ± 11.51 μm; the longest telopode width was 0.31 ± 0.17 μm, while the shortest was 0.05 ± 0.02 μm. The longest diameter of the podoms was 1.19 ± 0.53 μm, while the shortest was 0.37 ± 0.17 μm. The details of the above‐analysed parameters are listed in Table S1.

### 3.2 Distribution and spatial organization of CTs in the *Xenopus tropicalis* myocardium

**Figure 3:**
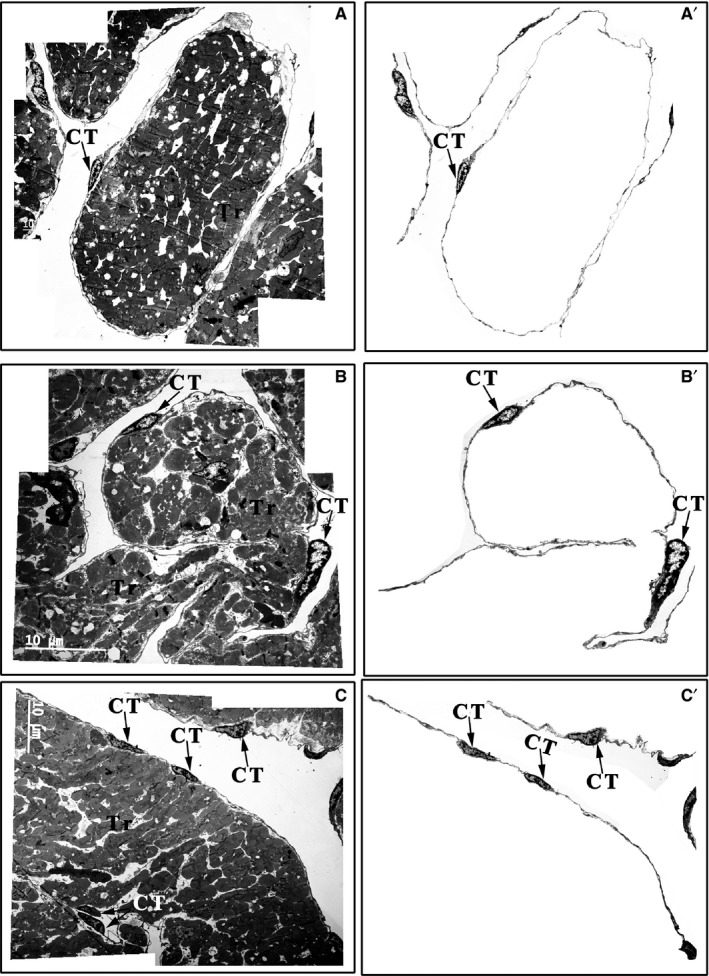
Distribution of CTs in the upper region, middle region and base of the *Xenopus tropicalis* myocardium. CTs mainly concentrated on the outer surface of trabeculae containing cardiomyocytes in the upper region (A), middle region (B) and base (C) of the *X tropicalis* myocardium. Most of the trabeculae are twined around one, two or several CTs and their telopodes or with telopodes alone. A′, B′ and C′: CTs of A, B and C that are not included in the trabecular structure; CT: Cardiac telocyte; Scale bar: Size as shown in the figures; Tr: Trabecula of the myocardium

CTs are mainly located on the outer surface of the trabeculae in the *X tropicalis* myocardium. With their cell body and telopodes, CTs closely connect with cardiomyocytes included in the trabeculae. Most of the trabeculae in the myocardium are twined around one to several CTs and their telopodes or around telopodes alone (Figure 3; Figure S4). In a given trabecula and among all trabeculae, CTs twined around the outer surface are able to connect using their telopodes (Figure 3C; Figure S4). These unique characteristics of distribution are similar among the upper region, middle region and base of the *X tropicalis* myocardium. Taking together the above evidence of the characteristic distribution of CTs in the three‐dimensional view indicates that CTs appear to be twined around the surface of the trabeculae and to be linked together as a three‐dimensional network in the *X tropicalis* myocardium (Figure 3; Figure S4).

### 3.3 Contacts of CTs

#### 3.3.1 Contacts between CT cell bodies and cardiomyocytes

The cell bodies of CTs did not contact or form a junction with cardiomyocytes. The average longest gap between the CT cell bodies and cardiomyocytes was 1.05 ± 0.78 μm, while the average smallest gap was 0.21 ± 0.20 μm (Table S2). Many microfilaments, arranged in a vertical and horizontal network with collagen, fill the gaps to link the CT cell bodies and cardiomyocytes (Figure S5A‐D).

#### 3.3.2 Contacts between CT telopodes and cardiomyocytes

**Figure 4:**
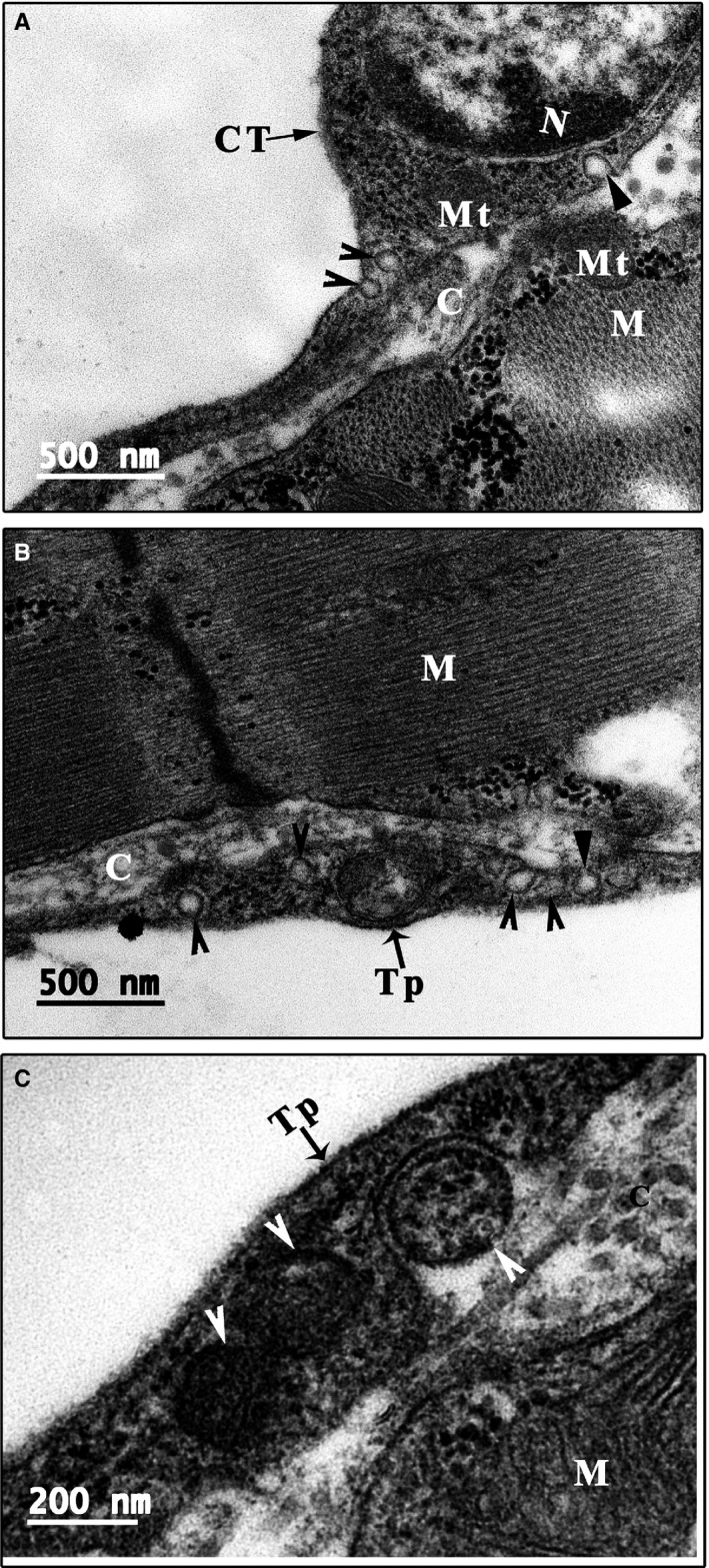
Vesicles and caveolae of CTs. Many vesicles (arrowhead) or coated vesicles (white arrowhead) are present in the telopodes. In addition, many caveolae are present in the membrane of the cell body and telopode (small triangle). The opening site of caveolae faces the extracellular space, and some vesicles or coated vesicles are located near the opening site of caveolae (A‐C). C: Collagen and extracellular matrix; CT: Cardiac telocyte; M: Cardiomyocyte; Mt: Mitochondria; N: Nucleus. Scale bar: Size as shown in the figures; Tp: Telopode

Similar to the CT cell body, the telopodes of the CTs did not directly contact or form a junction with cardiomyocytes (Figure S6). The mean longest gap between CT telopodes and cardiomyocytes was 1.59 ± 1.40 μm, while the mean smallest gap was 0.16 ± 0.26 μm (Table S2). Similarly, many microfilaments, arranged in a vertical and horizontal network with collagen, fill the gaps to link the CT telopodes and cardiomyocytes (Figure S6). In addition, there are many vesicles or coated vesicles in the telopodes and the gaps between long CT telopodes and cardiomyocytes (Figure 4).

#### 3.3.3 Contacts among CT telopodes

**Figure 5:**
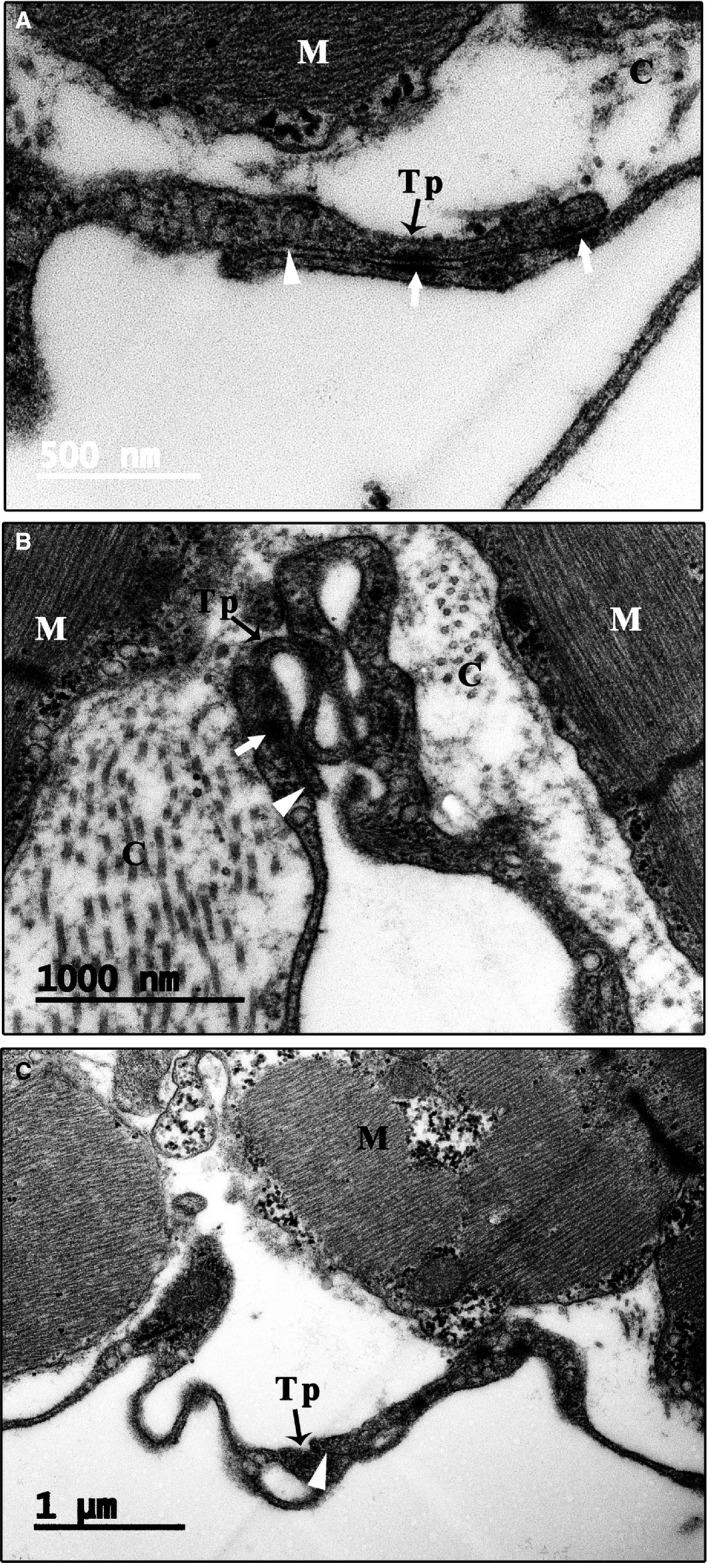
Telopode‐telopode contact. Most of the CTs link with other CTs via connections between the far ends of their telopodes (A‐C). Two types of telopode‐telopode contacts are observed: (1) a gap‐junction–like structure coming into nanometer‐range contact, in which some areas make nanometer‐range contact and other areas have a structure resembling one to two gap junctions (white arrow); (2) a nanometer‐range contact that does not have a well‐established junction at the far end of the telopodes in which two telopodes closely connect at a distance of approximately 1‐2 nm (white small triangle). Scale bar: Size as shown in the figures. C: Collagen and extracellular matrix; M: Cardiomyocyte; Tp: Telopode

Most CTs linked with other CTs via the far ends of their telopodes. Two types of telopode‐telopode contacts were observed: (a) a gap‐junction–like structure coming into nanometer‐range contact, in which some areas are in nanometer‐range contact and other areas have a structure resembling one or two gap junctions (Figure 5A); (b) a nanometer‐range contact that does not have a well‐established junction at the far end of the telopodes and in which two telopodes closely connect at a distance of approximately 1‐2 nm (Figure 5B,C).

#### 3.3.4 Contact between the CT cell body and the telopodes of other CTs

The CT cell body was able to make nanometer‐range contact with the telopodes of other CTs (Figure S5E,F). Distinct from the contacts between the far ends of telopodes from different CTs, telopodes from different CTs are able to form a nanometer‐range connection via a gap‐junction–like structure (Figure S5E,F).

### 3.4 Vesicles and caveolae of CTs

In the scarce cytoplasm of the CTs, some vesicles are present (Figure 1C; Figure 4A). One unique characteristic of CTs is the presence of many single vesicles or coated vesicles in the telopodes (Figures 1D,E, 4 and 5; Figures S6 and S7). The average longest diameter of the vesicles was 118.75 ± 15.93 nm, while the average smallest diameter was 98.14 ± 13.00 nm. In addition, the mean longest diameter of the coated vesicles was 244.37 ± 59.35 nm, and the average smallest diameter was 187.50 ± 56.35 nm (Table S2). The single vesicles are distributed along the telopode (Figure 4; Figure S6A,B), while many single vesicles and most of the coated vesicles are concentrated in the podoms of the telopode (Figures 1D and 4B,C). In addition, many caveolae are present in the membrane of the cell body and telopodes. The opening sites of the caveolae face the extracellular space, and some vesicles or coated vesicles are present around the opening site of the caveolae (Figure 4; Figure S7). In addition, the average diameter of the caveolae was 57.14 ± 18.75 nm (Table S2).

### 3.5 CTs recover more quickly than cardiomyocytes in the injured myocardium

**Figure 6:**
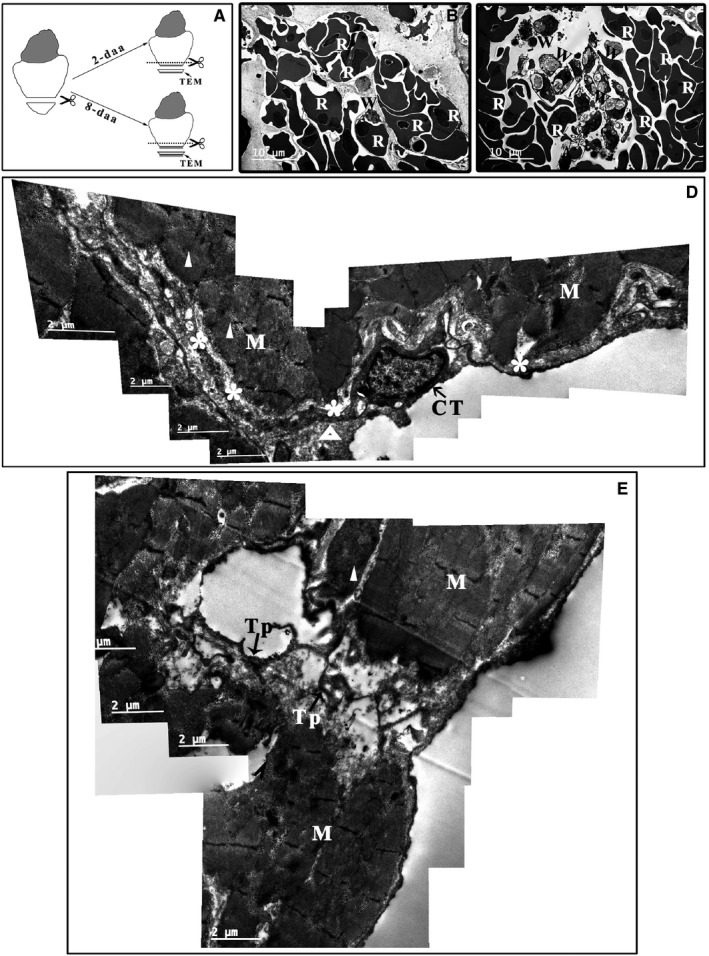
TEM analysis of the *Xenopus tropicalis* myocardium 2 d after injury. A, Schematics of the region of TEM analysis. Two days after amputation, red blood cells (B) and inflammatory cells (C) accumulate in the wound. Myofibres are disorganized in some cardiomyocytes on the border of the wound (white triangle), and disorganized telopodes of some CTs (white asterisk) are found. There are some clot structures in the extracellular space (open triangle) (D). In addition, some network structures consisting of disorganized telopodes and extracellular matrix tissue but lacking cardiomyocytes are present in the wound area (E). Scale bar: Size as shown in the figures. Asterisk: Disorganized telopode; C: Collagen and extracellular matrix; CT: Cardiac telocyte; M: Cardiomyocyte; Open triangle: Clot structure; R: Red blood cell; Tp: Telopode; W: Inflammatory cell; White Triangle: Disorganized myofibre

**Figure 7:**
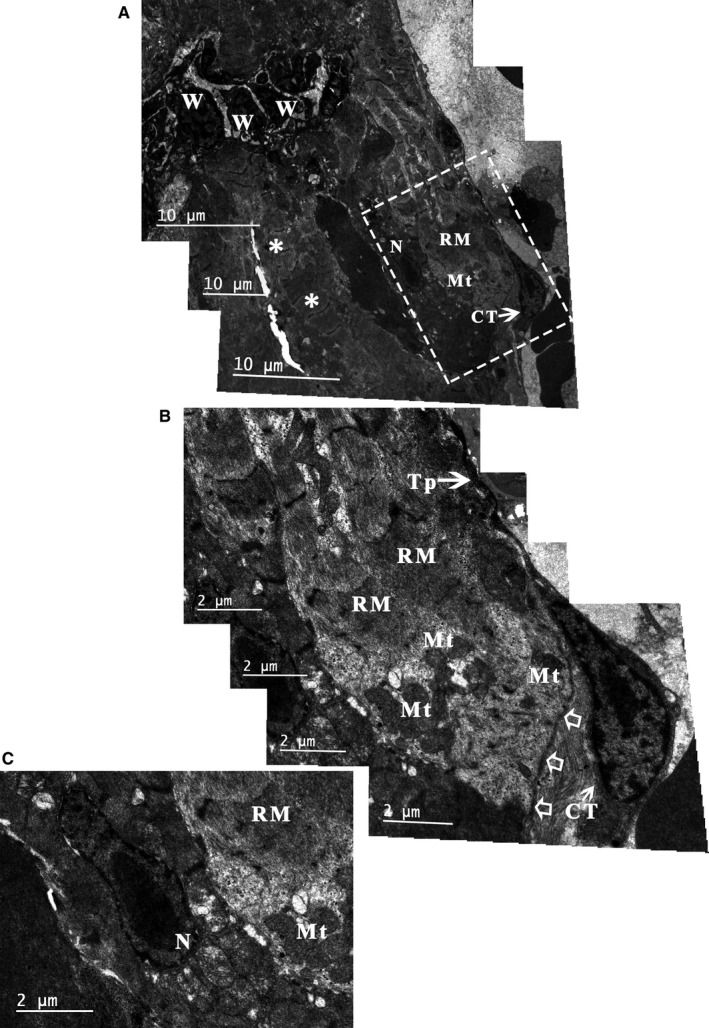
TEM analysis of the *Xenopus tropicalis* myocardium 8 d after injury. Eight days after injury, some of the injured muscle fibres regenerated via a novel muscle fibre characterized by an irregular muscle fibril arrangement and irregular sarcomere (RM) as well as regenerated sarcolemma (open arrow). An accumulation of mitochondria is seen in the regenerated muscle fibres (A, B), and a karyokinesis‐like nucleus was found in the border cardiomyocytes of regenerated myofibres (C). In addition, CTs with normal morphology are found on the outer surface of regenerated myofibres (A, B). B: Higher‐power view of the dotted line rectangle in A. Scale bar: Size as shown in the figures. Asterisk: Cardiomyocyte; CT: Cardiac telocyte; Mt: Mitochondria; N: Nucleus; RM: Regenerated myofibres; Tp: Telopode; W: Inflammatory cell

To investigate whether CTs are affected in the injured *X tropicalis* myocardium, approximately 10% of the apex was amputated, and the wound site was observed using TEM at 2 and 8 days. At 2 days, red blood cells (Figure 6B) and inflammatory cells (Figure 6C) accumulated in the wound. Myofibre disorganization was found in the border area of the wound (Figure 6D,E), and disorganized telopodes were found in some CTs (Figure 6D). Some clot structures were found in the extracellular space between the cardiomyocytes and CTs (Figure 6D). In addition, the wound area contained some network structures that consisted of disorganized telopodes and extracellular matrix tissue but lacked cardiomyocytes (Figure 6E). At 8 days, some injured muscle fibres regenerated via a novel muscle fibre characterized by an irregular muscle fibril arrangement and irregular sarcomeres as well as regenerated sarcolemma (Figure 7A,B). An accumulation of mitochondria was found in the regenerated muscle fibres, and a karyokinesis‐like nucleus was found in the border cardiomyocytes of regenerated myofibres (Figure 7). In contrast, CTs with normal morphology were found on the outer surface of regenerated myofibres (Figure 7A,B). All these findings suggested that 8 days after the cardiac resection, destructed CTs in the damaged myocardium were recovered, while the cardiomyocyte regeneration was not yet complete, the reconstruction of the CT network in the wound area might be an important step for initiating and maintaining the regeneration of injured myocardium and that mitochondria accumulation is needed in the regeneration of injured myocardium.

## 4 DISCUSSION

In the present study, we first showed that cardiac telocytes exist in the adult *X tropicalis* heart using TEM according to the presence of hallmark ultrastructural compartments of telocytes along with c‐Kit (maker for CTs) positivity and CD31 and vWF (unique markers for endothelial cells) negativity, which was confirmed by double immunofluorescent staining for anti–c‐Kit and anti‐CD31 or anti‐vWF. Based on the observations of the representative dimensions of the upper region, middle region and base of the ventricle of the *X tropicalis* heart, we found that CTs were mainly twined around the surface of cardiomyocyte trabeculae and were linked together via the ends of the telopodes, producing a three‐dimensional network of CTs in the *X tropicalis* myocardium. This distribution pattern was quite similar among the upper region, middle region and base region and was similar to that in other species, such as the leech, rodent and human.^5^, ^6^, ^25^, ^37^ This spatial distribution of CTs suggests that CTs and their networks play an important role as structural supports to maintain and promote the integrity of the *X tropicalis* myocardium under physiopathological conditions. In support of this hypothesis, our TEM observations revealed that even though the cell body and long telopodes of CTs do not make direct contact, the nanometer‐range gap between the CT cell body or telopodes and cardiomyocytes is spanned with microfilaments. This unique structural linkage justifiably fits the critical role of CTs in supporting cardiomyocytes and transducing mechanical stress on a given cardiomyocyte trabecula. These distributive characteristics are different from those of the mammalian heart, such as the hearts of humans and rodents (rats and mice). As in mammals, CTs show a distribution mainly in the gaps between cardiomyocytes.^5^, ^6^, ^25^, ^27^, ^28^, ^29^, ^37^ It is believed that this pattern fits the course of evolutionary development, as in the mammalian myocardium, cardiomyocytes do not have a trabecular structure like that in the amphibian *X tropicalis* myocardium.^41^, ^42^ The unique distribution of CTs in the *X tropicalis* myocardium also suggests that different from cardiomyocytes in the mammalian myocardium, cardiomyocytes in the myocardium of lower species, such as *X tropicalis*, might work together using trabeculae as structural and functional units to maintain cardiac function under physiopathological conditions. Meanwhile, CTs participate and might play an important role in cardiomyocyte communication and functional support for cardiomyocytes within a trabecula and across trabeculae. Indeed, our TEM observations revealed that most of the CTs are distributed on the outer surface of the trabeculae and that the CTs and telopodes within one trabecula are able to form nanocontacts with CTs and telopodes from other trabeculae.

In addition, TEM analysis showed that many microvesicles and coated vesicles are present in the long telopodes of most of the CTs. The coated vesicles were mainly concentrated in the podoms, while single vesicles were mainly located in the podomeres. Some microvesicles were present in the CT cell body, while others as well as some coated vesicles were found on outside of the cell body, podom or podomere. This morphological evidence implies that CTs might be able to communicate with the nursed cell (such as a cardiomyocyte) via microvesicles secreted from the CT cell body and telopodes. In addition, it is generally recognized that coated vesicles play an important role in receptor‐mediated endocytosis.^43^, ^44^, ^45^ However, the design of the present study fails to answer the questions regarding the exact functional role and the related mechanism of microvesicles and coated vesicles found in CTs. Therefore, further functional study of CT‐derived microvesicles and coated vesicles as well as an investigation of the possible differences in cargoes among microvesicles and coated vesicles might uncover the function of CTs and the underlying means of CT‐cardiomyocyte communication.

Our TEM analysis of damaged *X tropicalis* myocardium revealed some network structures that consisted of disorganized telopodes and extracellular matrix tissues but lacked cardiomyocytes in the wound site 2 days after myocardial injury. Moreover, at 8 days after injury, novel regenerated nonmature myofibres accompanied by the accumulation of mitochondria were found in the wound site, and a karyokinesis‐like nucleus was found in the border cardiomyocytes of regenerated myofibres. In addition, CTs with normal morphology were located on the outer surface of regenerated myofibres. Taking into consideration the findings of our previous report with the same animal model wherein the adult damaged *X tropicalis* myocardium is able to regenerate in a scar‐free manner,^36^ the present results further indicated that reconstruction of the CT network in the wound area might be an important step for initiating and maintaining the regeneration of injured myocardium and that mitochondria accumulation is needed in the regeneration of injured myocardium.

In summary, the present ultrastructural results clearly indicate that CTs exist in the *X tropicalis* myocardium. CTs in the *X tropicalis* myocardium were mainly twined around the surface of cardiomyocyte trabeculae and were linked together via nanocontacts between the ends of telopodes, forming three‐dimensional networks. The cardiomyocytes in the *X tropicalis* myocardium might function with CTs in a regulated microenvironment using trabeculae as structural and functional units to maintain cardiac function under physiopathological conditions. The regeneration of CTs might be a critical step in initiating and maintaining the regeneration of damaged myocardium in *X tropicalis* and potentially in mammals, including humans.

## CONFLICTS OF INTEREST

The authors confirm that there are no conflicts of interest.

## AUTHOR CONTRIBUTIONS

LL, ZL, JL, HC, HG and JY performed most of the experiments and analysed data; RH, QP, HZ, ZY, SF and XQ contributed to discussion and manuscript writing; DC conceived and designed this work and wrote the manuscript.

## Data Availability

The data that support the findings of this study are available on request from the corresponding author.
