# Supplementary figures and images for: Cardiac telocytes exist in the adult Xenopus tropicalis heart
Source: J Cell Mol Med. 2020 Jan 12;24(4):2531–41. doi: 10.1111/jcmm.14947 (PMC7028868; doi:10.1111/jcmm.14947)

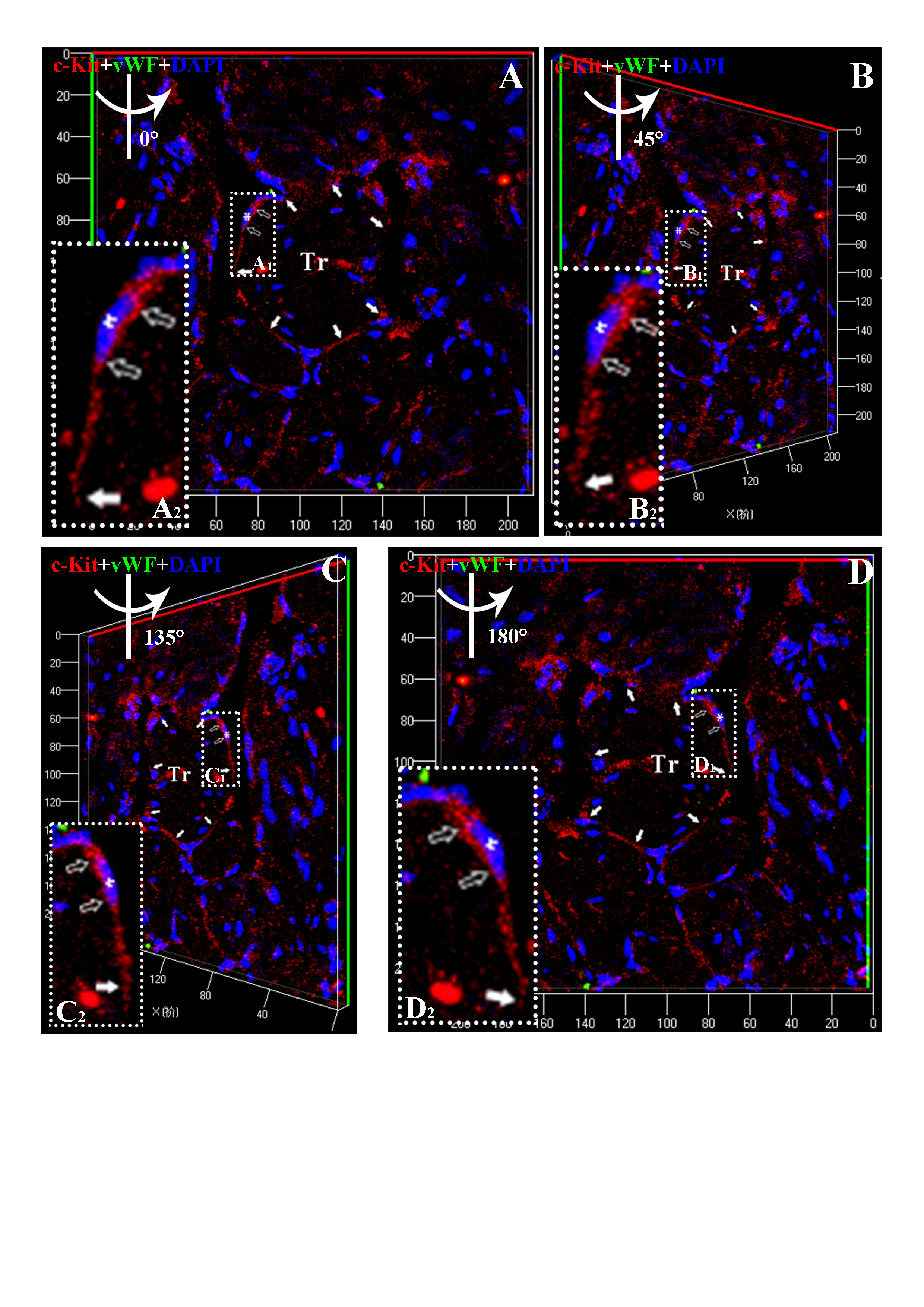

Supplement: Supplementary file 1 [file JCMM-24-2531-s001.tif]

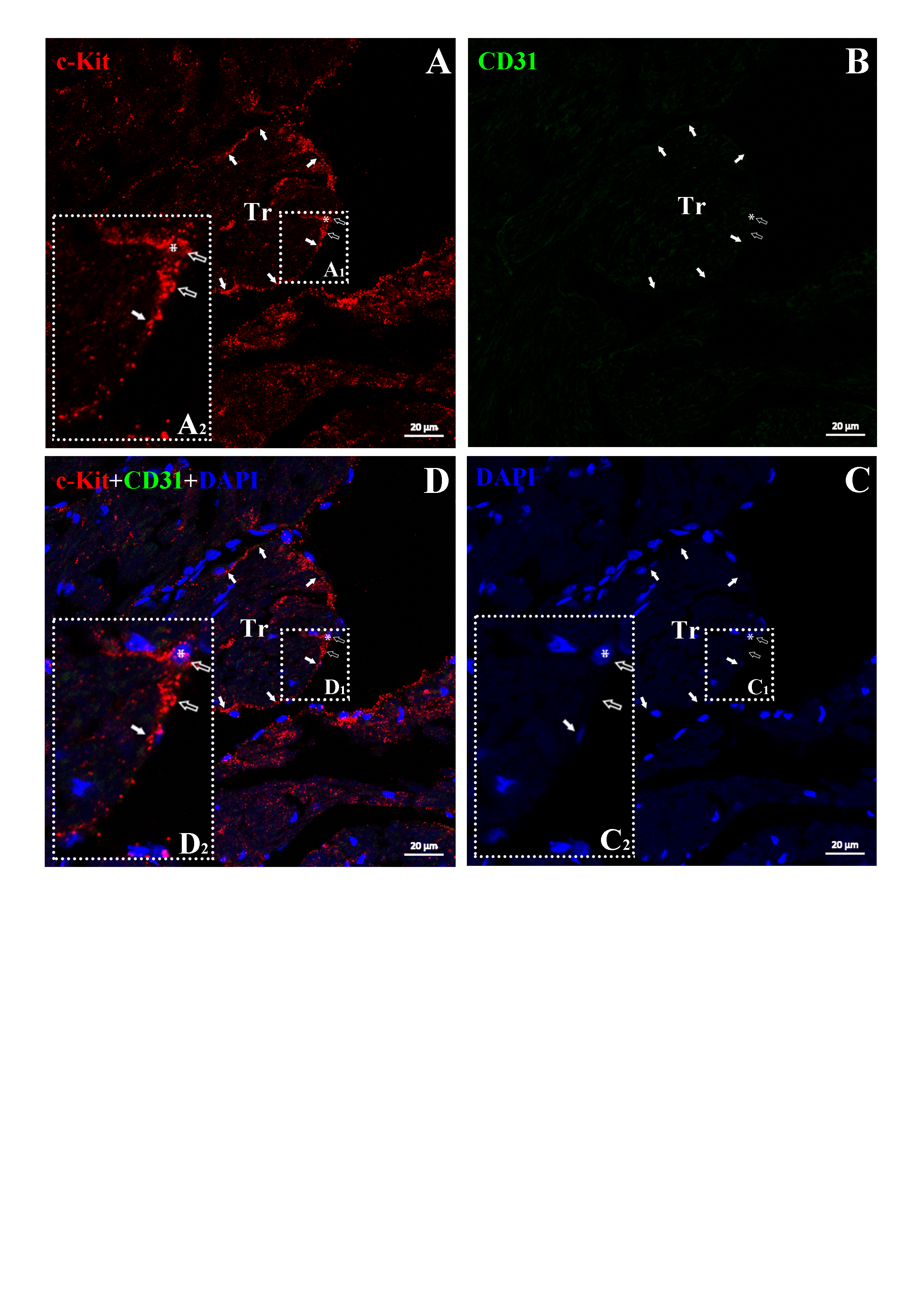

Supplement: Supplementary file 2 [file JCMM-24-2531-s002.tif]

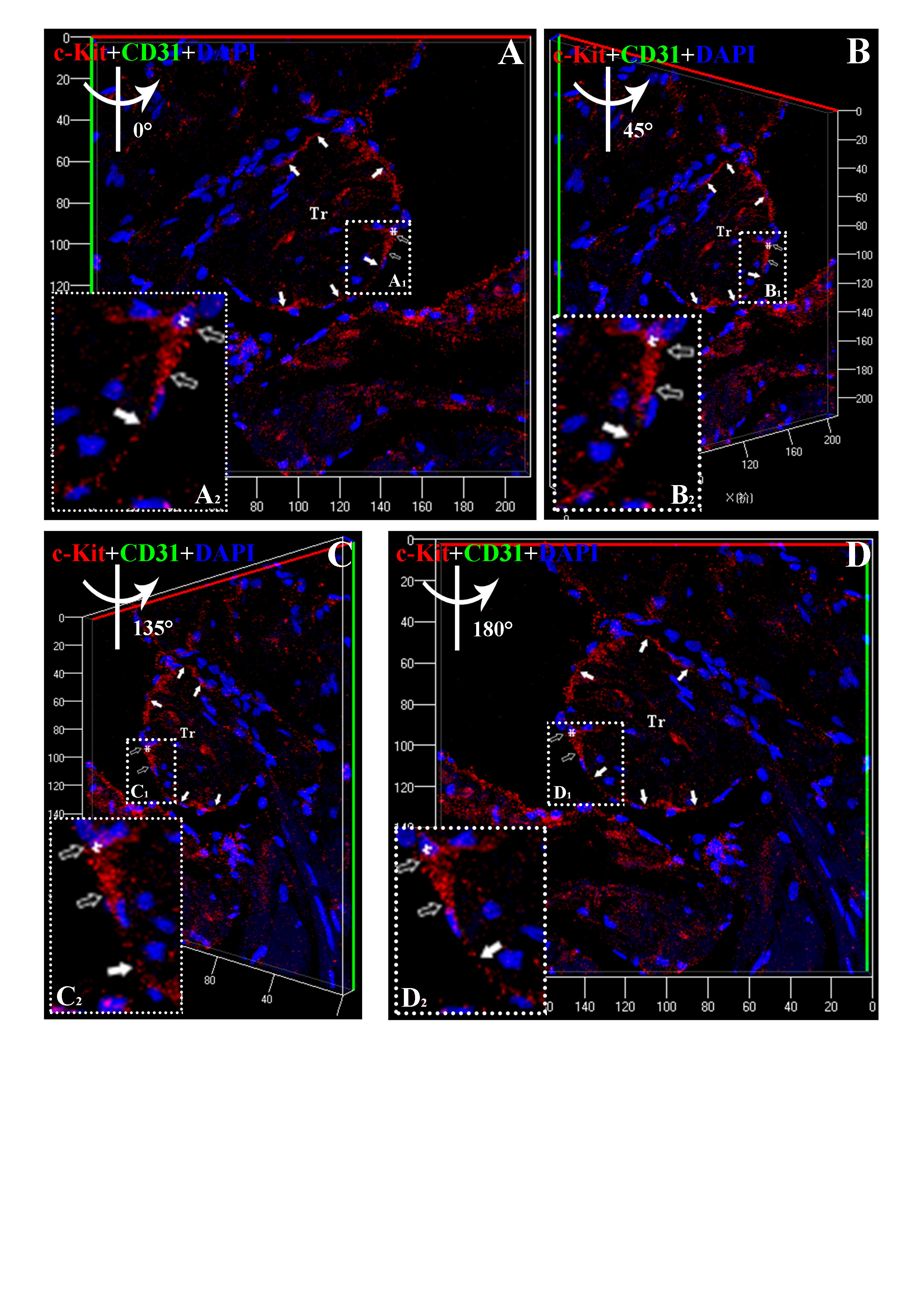

Supplement: Supplementary file 3 [file JCMM-24-2531-s003.tif]

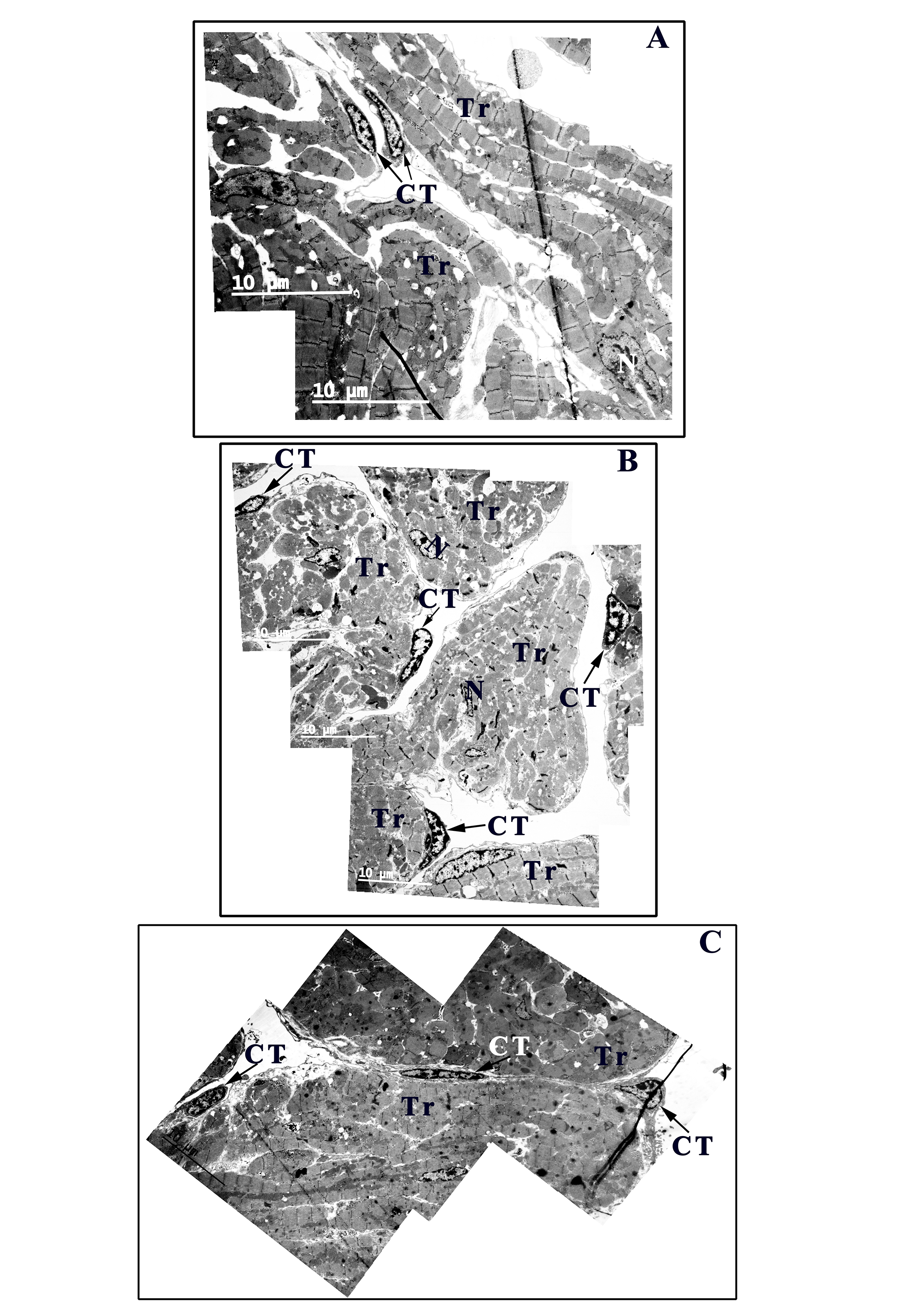

Supplement: Supplementary file 4 [file JCMM-24-2531-s004.tif]

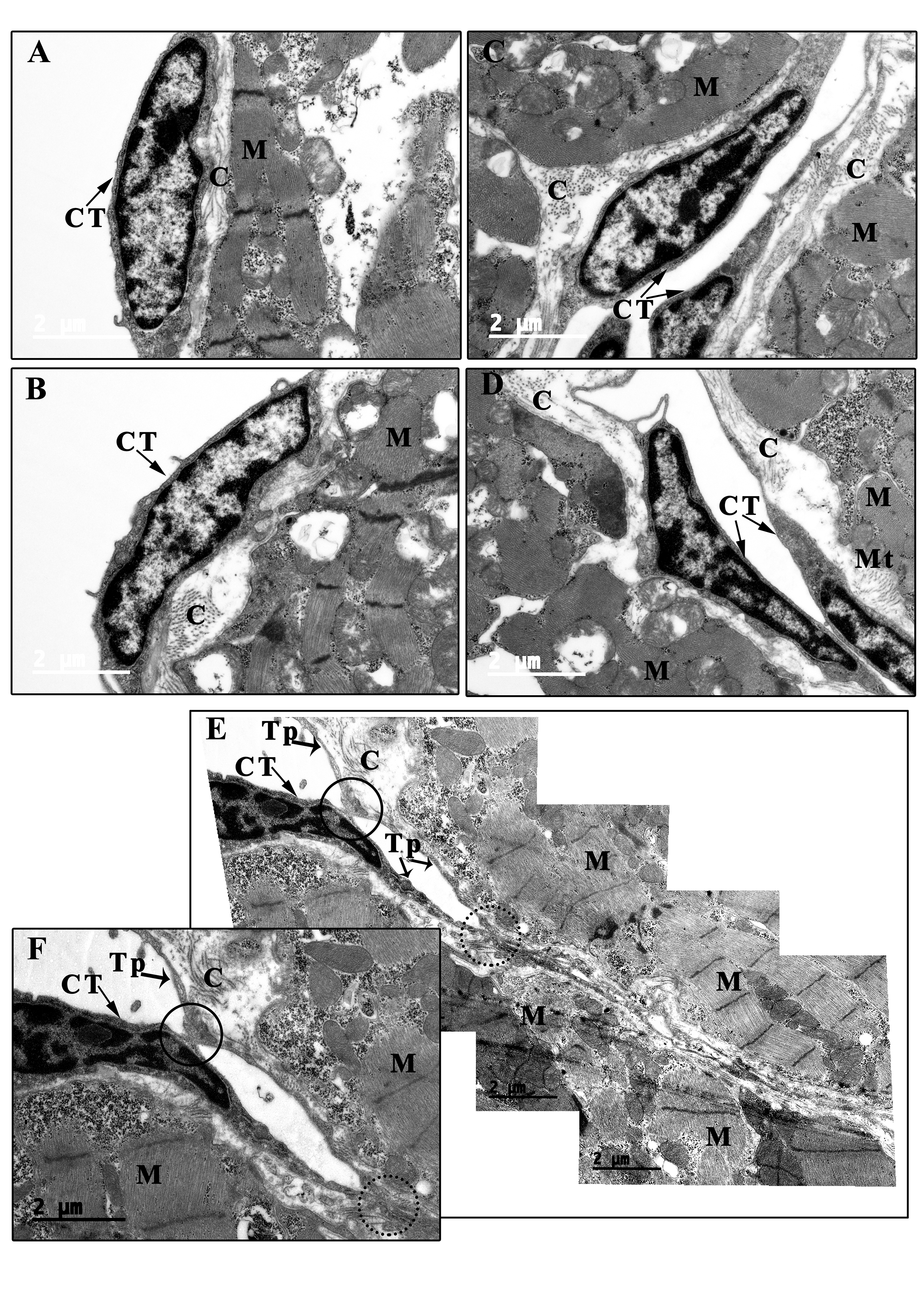

Supplement: Supplementary file 5 [file JCMM-24-2531-s005.tif]

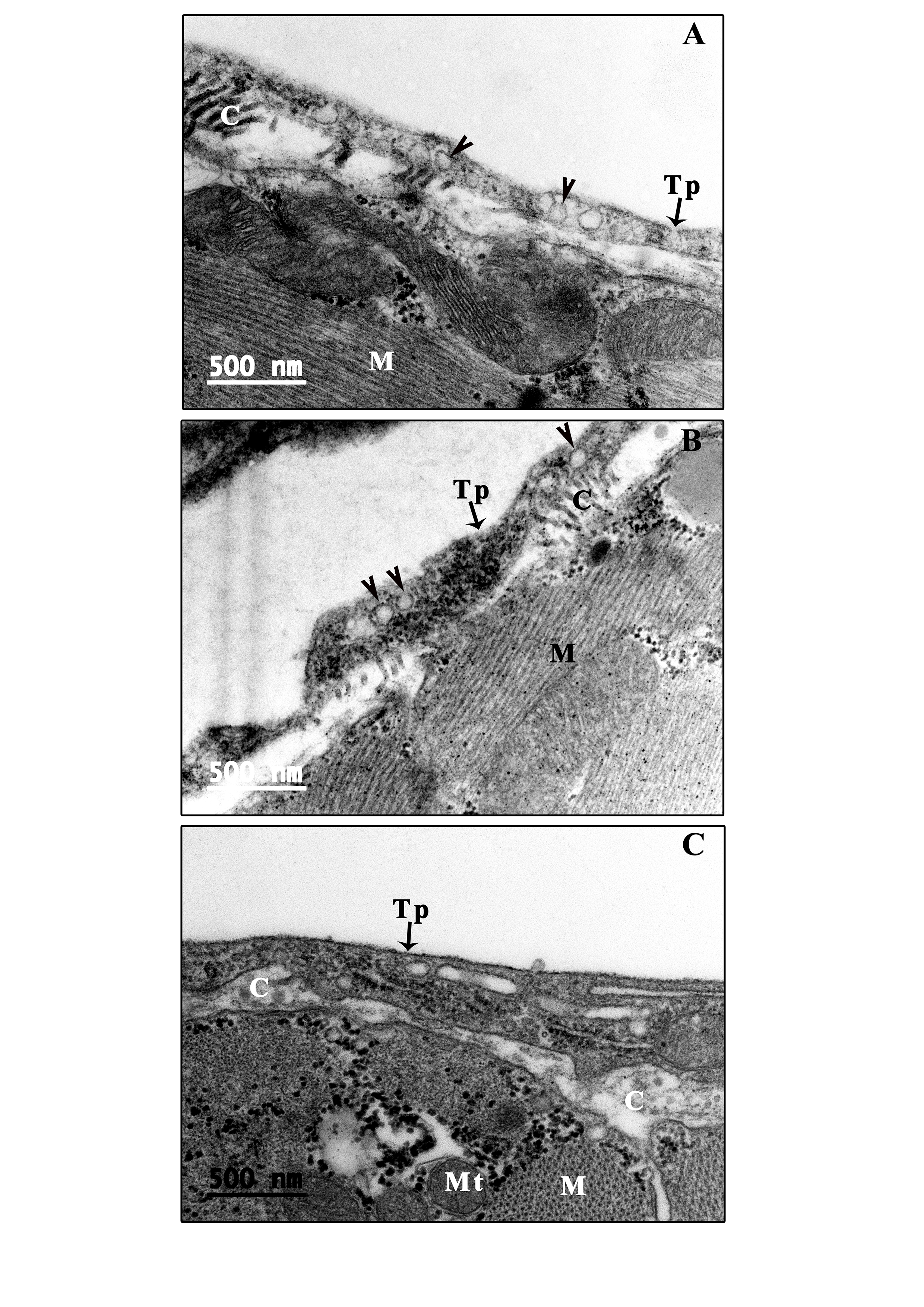

Supplement: Supplementary file 6 [file JCMM-24-2531-s006.tif]

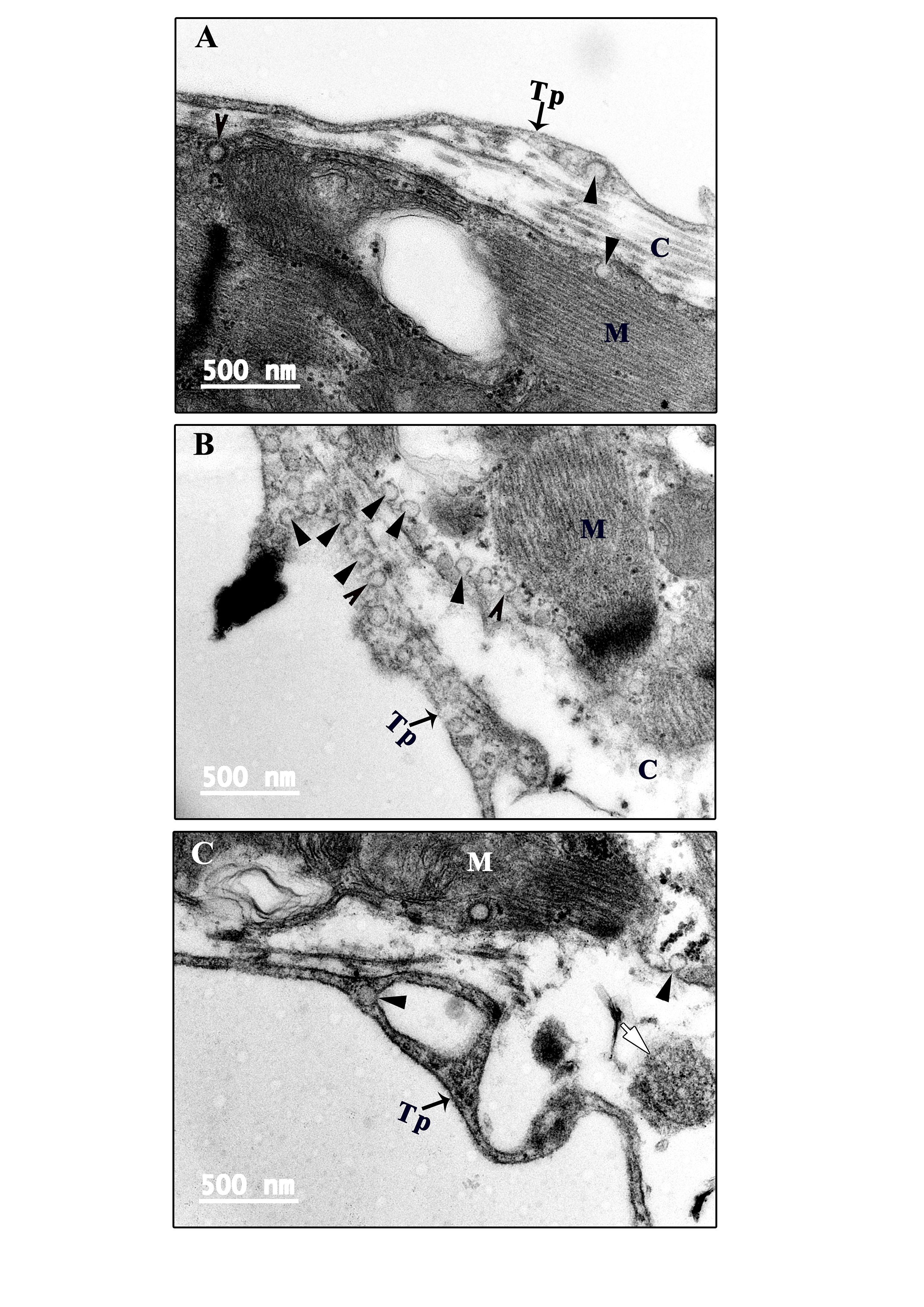

Supplement: Supplementary file 7 [file JCMM-24-2531-s007.tif]
