# Supplementary material for: Cardiac telocytes exist in the adult Xenopus tropicalis heart
Source: J Cell Mol Med. 2020 Jan 12;24(4):2531–41. doi: 10.1111/jcmm.14947 (PMC7028868; doi:10.1111/jcmm.14947)
Supplement: Supplementary file 8 [file JCMM-24-2531-s008.docx]

**Supporting Information**

**Table S1: Semiquantitative analysis of CTs in the *X. tropicalis* myocardium (I)**

|  |  |  |  | **The dimension of the ventricle** | | |  |
| --- | --- | --- | --- | --- | --- | --- | --- |
| **Measurement** | **Mean±SD** | **Min** | **Max** | **Upper** | **Middle** | **Base** | **Number** |
| The longest diameter of the CT cell body (μm) | 7.68±1.82 | 3.24 | 11.95 | 25 | 35 | 7 | 67 |
| The shortest diameter of the CT cell body (μm) | 2.00±0.64 | 0.86 | 3.84 | 25 | 35 | 7 | 67 |
| The longest diameter of the nucleus (μm) | 5.84±1.73 | 1.44 | 9.09 | 25 | 35 | 7 | 67 |
| The shortest diameter of the nucleus (μm) | 1.64±0.60 | 0.55 | 3.03 | 25 | 35 | 7 | 67 |
| The ratio of the area of the nucleus to the area of the cell body of CTs | 0.64±0.14 | 0.24 | 0.88 | 24 | 35 | 7 | 66 |
| The length of telopodes (μm) | 19.65±11.51 | 3.33 | 61.5 | 20 | 26 | 3 | 49 |
| The longest telopode width (μm) | 0.31±0.17 | 0.10 | 0.88 | 23 | 29 | 3 | 55 |
| The shortest telopode width (μm) | 0.05±0.02 | 0.02 | 0.11 | 23 | 31 | 3 | 57 |
| The longest diameter of the podoms (μm) | 1.19±0.53 | 0.45 | 2.82 | 21 | 21 | 3 | 45 |
| The shortest diameter of the podoms (μm) | 0.37±0.17 | 0.17 | 0.91 | 21 | 21 | 3 | 45 |

Upper: The counting number of the upper region of ventricle. Middle: The counting number of the middle region of ventricle. Base: The counting number of the base region of ventricle. Number: The total counting number of the upper, middle and base region of ventricle.

**Table S2: Semiquantitative analysis of CTs in the *X. tropicalis* myocardium (II)**

|  |  |  |  | **The dimension of the ventricle** | | |  |
| --- | --- | --- | --- | --- | --- | --- | --- |
| **Measurement** | **Mean±SD** | **Min** | **Max** | **Upper** | **Middle** | **Base** | **Number** |
| The average longest gap between the CT cell body and cardiomyocytes (μm) | 1.05±0.78 | 0.26 | 4.60 | 23 | 32 | 7 | 62 |
| The average smallest gap between the CT cell body and cardiomyocytes (μm) | 0.21±0.20 | 0.03 | 0.81 | 19 | 18 | 3 | 40 |
| The mean longest gap between the CT telopodes and cardiomyocytes (μm) | 1.59±1.40 | 0.31 | 7.90 | 20 | 30 | 3 | 53 |
| The mean smallest gap between the CT telopodes and cardiomyocytes (μm) | 0.16±0.26 | 0 | 1.25 | 23 | 23 | 2 | 48 |
| The average longest diameter of vesicles (nm) | 118.75±15.93 | 86.23 | 159.27 | 36 | 41 | 5 | 82 |
| The average smallest diameter of vesicles (nm) | 98.14±13.00 | 75.30 | 135.02 | 36 | 41 | 5 | 82 |
| The mean longest diameter of coated vesicles (nm) | 244.37±59.35 | 158.91 | 359.00 | 8 | 9 | 2 | 19 |
| The average smallest diameter of coated vesicles (nm) | 187.50±56.35 | 78.93 | 297.88 | 8 | 9 | 2 | 19 |
| The average diameter of caveolae (nm) | 57.14±18.75 | 33.23 | 113.12 | 15 | 10 | 6 | 31 |

Upper: The counting number of the upper region of ventricle. Middle: The counting number of the middle region of ventricle. Base: The counting number of the base region of ventricle. Number: The total counting number of the upper, middle and base region of ventricle.
