# Supplementary material for: Cardiac telocytes exist in the adult Xenopus tropicalis heart
Source: J Cell Mol Med. 2020 Jan 12;24(4):2531–41. doi: 10.1111/jcmm.14947 (PMC7028868; doi:10.1111/jcmm.14947)
Supplement: Supplementary file 9 [file JCMM-24-2531-s009.docx]

**Supporting Information**

**Fig. S1:** **Three-dimensional reconstruction of Figure 2D****. A:** Three-dimensional reconstruction of Figure 2 in Z-axis. B: 45° rotation of A in y‐axis. C: 135° rotation of A in y‐axis. D: 180° rotation of A in y‐axis. Showing CTs (c-Kit positive) is around the trabeculae of *X. tropicalis* myocardium (white arrow) and the representative cell body (open arrow) and part of telopode of CTs (white arrow) in higher magnification (A_2_-D_2_) of selected area (white dot line rectangle; A_1_-D_1_) respectively. Red: anti-c-Kit; Green: anti-vWF; Blue: DAPI. Tr: Trabecula of the myocardium.

**Fig. S2: TEM identified CTs in myocardium is c-Kit positive but CD31 negative.** Double immuofluorescent staining for anti‐c‐Kit (red) and CD31 (green) demonstrated c‐Kit^+^ and CD31^-^ cells with very small cell bodies (open arrow), a nucleus (approximately 1:1 ratio of the cytoplasm to the nucleus; white asterisk) and extremely thin prolongation (telopode) around trabeculae in the *X. tropicalis* myocardium (white arrow). Showing that TEM identified CTs express c-Kit, a generally accepted marker of CTs, but not CD31, a unique marker of endothelial cell. A: Anti‐c‐Kit (red); B: Anti‐CD31 (green); C: DAPI; D: merged of A, B and C. Scale bar: 20μm. Tr: Trabecula of the myocardium.

**Fig. S3:** **Three-dimensional reconstruction of Figure S2D.** **A:** Three-dimensional reconstruction of Figure S2 in Z-axis. B: 45° rotation of A in y‐axis. C: 135° rotation of A in y‐axis. D: 180° rotation of A in y‐axis. Showing CTs (c-Kit positive) is around the trabeculae of *X. tropicalis* myocardium (white arrow) and the representative cell body (open arrow) and part of telopode of CTs (white arrow) in higher magnification (A_2_-D_2_) of selected area (white dot line rectangle; A_1_-D_1_) respectively. Red: anti-c-Kit; Green: anti-vWF; Blue: DAPI. Tr: Trabecula of the myocardium.

**Fig. S4: Distribution of CTs** **among trabeculae in the *X. tropicalis* myocardium.** Among the trabeculae, CTs twined around the outer surface are able to connect using their telopodes in the upper region (A), middle region (B) and base (C). Scale bar: Size as shown in the figures. CT: Cardiac telocyte. Tr: Trabecula of myocardium.

**Fig. S5: Contact between CTs and cardiomyocytes.** The CT cell body does not contact or form a junction with cardiomyocytes. Many microfilaments that are arranged as a vertical and horizontal network with collagen fill the gap to link the CT cell body with cardiomyocytes (A-F). In addition, the cell body of one CT is able to closely connect with the cell body of another CT (D), and the cell body of one CT is able to form a nanometer-range connection with the telopode of another CT (E, F; circle). Distinct from contacts between the far ends of telopodes from different CTs, two telopodes from different CTs are able to form a nanometer-range connection via a gap-junction-like structure (E, F; dotted line circle). Scale bar: Size as shown in the figures. CT: Cardiac telocyte. C: Collagen and extracellular matrix. Mt: Mitochondria. M: Cardiomyocyte. Tp: Telopode.

**Fig. S6: Contact between CT telopodes and cardiomyocytes.** The telopodes of CTs do not directly contact or form a junction with cardiomyocytes (A-C). Many microfilaments that are arranged as a vertical and horizontal network with collagen fill the gap to link the telopodes with cardiomyocytes (A-C). Many vesicles (arrow head) are present in the telopodes. Scale bar: Size as shown in the figures. C: Collagen and extracellular matrix. Mt: Mitochondria. M: Cardiomyocyte. Tp: Telopode.

**Fig. S7: Vesicles and caveolae of CTs and cardiomyocytes.** Some vesicles are present under membrane of the cardiomyocyte (A; arrow head). Many single vesicles are concentrated in the podoms of the telopode (B). In addition, many caveolae are present in the membrane of the cell body and the telopodes (A-C; small triangle). The opening site of caveolae faces the extracellular space, and some vesicles or coated vesicles (C; open arrow) are located around the opening site of caveolae. Scale bar: Size as shown in the figures. C: Collagen and extracellular matrix. M: Cardiomyocyte. Tp: Telopode.
